# Identification of the Optimal Model for the Prediction of Diabetic Retinopathy in Chinese Rural Population: Handan Eye Study

**DOI:** 10.1155/2022/4282953

**Published:** 2022-11-16

**Authors:** Shanshan Jin, Xu Zhang, Hanruo Liu, Jie Hao, Kai Cao, Caixia Lin, Mayinuer Yusufu, Na Hu, Ailian Hu, Ningli Wang

**Affiliations:** Beijing Institute of Ophthalmology, Beijing Tongren Eye Center, Beijing Tongren Hospital of Capital Medical University, Hougou Lane No 17, Chongnei Street, Beijing 100005, China

## Abstract

**Background:**

To identify an optimal model for diabetic retinopathy (DR) prediction in Chinese rural population by establishing and comparing different algorithms based on the data from Handan Eye Study (HES).

**Methods:**

Five algorithms, including multivariable logistic regression (MLR), classification and regression trees (C&RT), support vector machine (SVM), random forests (RF), and gradient boosting machine (GBM), were used to establish DR prediction models with HES data. The performance of the models was assessed based on the adjusted area under the ROC curve (AUROC), sensitivity, specificity, and accuracy.

**Results:**

The data on 4752 subjects were used to build the DR prediction model, and among them, 198 patients were diagnosed with DR. The age of the included subjects ranged from 30 to 85 years old, with an average age of 50.9 years (SD = 3.04). The kappa coefficient of the diagnosis between the two ophthalmologists was 0.857. The MLR model revealed that blood glucose, systolic blood pressure, and body mass index were independently associated with the development of DR. The AUROC obtained by GBM (0.952), RF (0.949), and MLR (0.936) was similar and statistically larger than that of CART (0.682) and SVM (0.765).

**Conclusions:**

The MLR model exhibited excellent prediction performance and visible equation and thus was the optimal model for DR prediction. Therefore, the MLR model may have the potential to serve as a complementary screening tool for the early detection of DR, especially in remote and underserved areas.

## 1. Introduction

Diabetic retinopathy (DR) is a common and serious microvascular complication triggered by diabetes. It is the leading cause of visual impairment and blindness among working age adults [1]. According to the World Report on Vision published by the World Health Organization (WHO) in 2020, at least 2.2 billion people have a vision impairment worldwide. In at least 1 billion—or almost half—of these cases, vision impairment could have been prevented or has yet to be addressed. Meanwhile, moderate or severe distance vision impairment or blindness in 3 million people was caused by diabetic retinopathy [2]. There were 92.4 million diabetic patients in China [3], and among them, 15.2 million developed DR [4]. The number of patients suffering from diabetes and related complications is continuously rising in China. Diabetic mellitus and DR have been major public health issues in China. Moreover, early symptoms like microaneurysm, retinal hard exudates, and cotton version are often insidious and hard to identify macroscopically. Early detection and management of DR can effectively prevent visual deterioration. Further, it has been verified that DR is also a risk factor for other diabetic complications onset like cardiovascular ones [5]. Thus, it is critical to determine the risk factors and the combined effects of these risk factors on DR.

By far, many factors such as age, body mass index (BMI), smoking status, duration of diabetes, glycemic control, systolic blood pressure (SBP), serum lipids, urinary albumin, and C peptide have been identified as risk factors for the development of DR via both cross-sectional and cohort prospective studies [6–9]. Advances in statistical methodology have provided the tools to model linear or nonlinear relationships among risk factors for specific diseases. Logistic regression is a well-established classification technique that is widely used in clinical studies [10]. However, its capacity for the prediction of nonlinear relationships among risk factors is limited. In recent years, many researchers switched their focus to machine learning field. Classic machine learning models like decision tree, neural network algorithm, and random forest method are widely used for the classification of diseases. There were several studies based on the machine learning model for the early detection of DR [11–13]. Given the difference in the algorithms and application condition of these machine learning models, the optimal model for the prediction of various diseases would differ. Here, we aim to apply and compare the prediction performance of multivariable logistic regression and machine learning algorithms based on the data of Handan Eye Study (HES) and to develop the practical classifier for DR identification among the Chinese rural population.

## 2. Subjects and Examination

**Figure 1:**
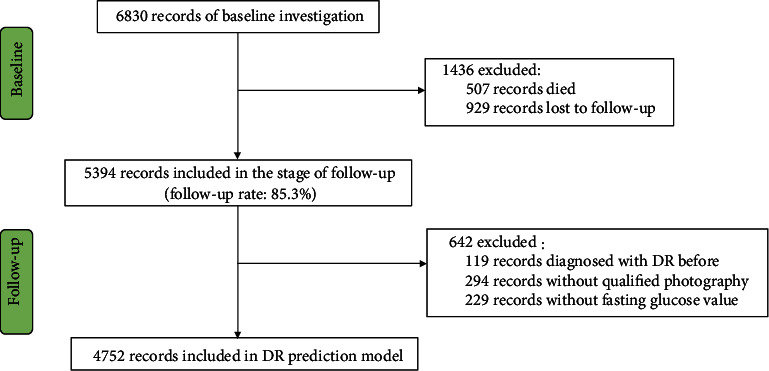
Flow chart of subject inclusion.

The investigational site was in Yongnian County of Handan City. The statistic comparison revealed that the gender and age distribution of Yongnian county were similar to those of the nationwide rural areas. To investigate the 6-year cumulative incidence of RD in HES [14], the follow-up study was conducted from 2012 to 2013 [15]. At baseline, there were 6830 participants [16], and among them, 5394 participated in the follow-up investigation. The follow-up rate was 85.3% (5394/6323) [15]. After excluding participants who were previously diagnosed or lacked qualified fundus photography or data on fasting glucose value, data from 4752 participants were collected to build the DR prediction model (Figure 1).

All study subjects underwent standardized and comprehensive physical and ophthalmic examinations. The physical examination included height, weight, BMI, SBP and diastolic blood pressure (DBP), and other physical function measurements. The ophthalmic examinations covered the following items: (1) autorefraction and visual acuity (VA) measurement [17], (2) visual field testing, (3) intraocular pressure (IOP) measurement [18], (4) slit-lamp examination, (5) fundus photography, (6) anterior segment optical coherence tomography, (7) optic disc imaging, (8) retinal nerve fiber layer imaging, (9) ocular biometry, and (10) gonioscopy. In addition, the blood and urine samples were collected, for which, the data were generated by the examination machine automatically.

### 2.1. Diagnosis of DR

The diabetic patients who were diagnosed with DR at the baseline investigation were excluded. Therefore, the cases diagnosed during this follow-up period were the new cases that developed between the year of 2007 and the year of 2012. Two qualified ophthalmologists (Ailian Hu and Xu Zhang) reviewed the fundus photographs independently, and the diagnosis was made according to the ETDRS. The kappa coefficient was calculated to evaluate the consistency of the diagnosis between the two ophthalmologists.

### 2.2. Model Introduction

Multivariable logistic regression (MLR) is a well-established classification technique widely used in epidemiological studies, which is constructed based on maximum likelihood estimation. Apart from the logistic regression, four machine learning algorithms were used to establish the prediction model on DR in HES.

#### 2.2.1. Classification and Regression Trees (C&RT)

C&RT is a type of nonparametric decision tree methodology that was first developed by Breiman et al. [19] and has been widely applied to clinical and public health researches [20–23]. C&RT analysis is a nonparametric decision tree methodology that produces a visual output that is a multilevel structure that resembles the branches of a tree. C&RT analysis set of if-then conditions permits the classification of cases and then efficiently segments populations into meaningful subgroups. C&RT has the advantage of ignoring irrelevant descriptors and handling multiple mechanisms of action.

#### 2.2.2. Support Vector Machine (SVM)

SVM are linear classifiers based on the margin maximization principle. SVM accomplishes the classification task through the following steps: SVM maps the data into a higher dimensional input space and then constructs an optimal separating hyperplane in this space. The hyperplane has the ability to optimally separate the data into two regions, each of which is also called a class two categories [24, 25]. The SVM is capable of dealing with high-dimensional data and has excellent generalization performance. However, it is not robust to the presence of a large number of irrelevant descriptors, thus requiring descriptor preselection as well.

#### 2.2.3. Random Forests (RF)

RF belongs to “ensemble learning” proposed by Breiman and Quinlan in 2001 [26]. Ensemble learning generates many classifiers and aggregates the results of these classifiers. Boosting [27] and bagging algorithms [26] are well-known methods of ensemble learning. RF is a typical machine learning model of bagging algorithms. It draws multiple samples from the original sample based on the Bootstrap replicated sample method and constructs a decision tree model for each Bootstrap sample, then accomplishes the classification task by scored and averaged predict results of these decision tree models. RF has a high prediction accuracy, good tolerance to outliers and noise, and less probability of model overfitting [28–30]. Therefore, it has been widely applied in the fields of medicine, bioinformatics, and economics [31–34].

#### 2.2.4. Gradient Boosting Machine (GBM)

GBM is a typical machine learning model of boosting algorithms and was proposed by Friedman in 2001 [35]. The learning procedure of GBM is consecutively fitting new models, which is a process of consecutive iteration. Subsequently, it would be able to provide a more accurate estimate of the response variable. The purpose of each iteration is to reduce the residual generated by the previous iteration. According to the Newton-Raphson method, the new model will be constructed based on the orientation of the gradient-descent of the previous residual [36]. To avoid the over-fitting of the model, the GBM introduced the shrinkage parameter which is related to the learning ability of the model. Therefore, GBM has excellent generalization performance [37].

### 2.3. Statistical Analysis

All the statistical analyses were performed with an open-source R-program (version 3.6.2). The mean value and standard deviation were used for the statistical description of continuous variables, and frequency and percentage were used for the statistical description of categorical variables. Continuous variables were analyzed by Student's *t*-test or Wilcoxon rank sum test, and categorical variables were analyzed by chi-squared test or rank sum test. The odds ratio (OR) value of each variable with the corresponding 95% confidence interval (95% CI) was calculated. In the present study, 70% of samples (train set) were applied to construct the prediction models, while the rest of the samples, namely the validation set, was applied to estimate the prediction performance of these models. Receiver operating characteristic (ROC) curve was adopted for the evaluation of the prediction performance of the model. The ROC curve was plotted according to the prediction probability values obtained by the model. The area under the ROC curve was compared by the *Z* test. The statistic *P* value less than 0.05 was considered to be significant. The prediction probability threshold is set at the corresponding value of Youden's index (YI) on the ROC curve, and the prediction probability of any subject greater than the threshold indicates the development of DR.

## 3. Results

### 3.1. Demographic Characteristics and Univariate Analysis

Among 4752 subjects included in the current study, 46.6% were male and 53.3% were female. The age of the included subjects ranged from 30 to 85 years old, with an average age of 50.9 years (SD = 3.04). A total of 198 patients were diagnosed with DR. After the univariate analysis, 9 variables were extracted as the impact factors of the DR. The results of univariate analysis on the demographic characteristics are shown in Table 1.

### 3.2. The Kappa Coefficient between Two Ophthalmologists

As shown in Table 2, the kappa coefficient was 0.857 which was indicating a good consistency between these two ophthalmologists.

### 3.3. Performance of MLR

**Figure 2:**
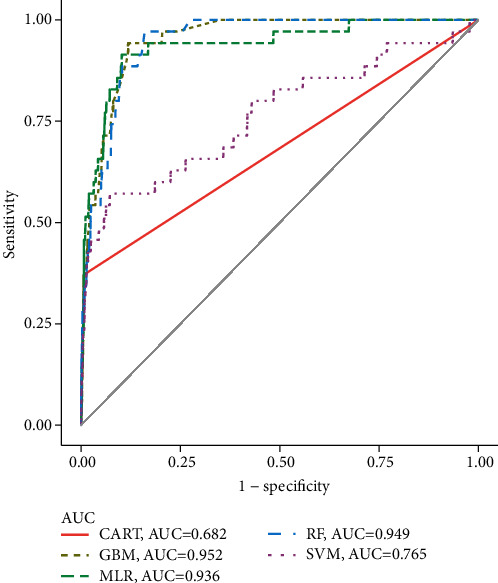
ROC curve of prediction models.

The MLR model showed that blood glucose, SBP, and BMI were independently associated with the development of DR. The results are shown in Table 3. The ROC curve obtained by logistic regression is presented in Figure 2. The adjusted area under ROC curve (AUROC) was 0.936. As shown in Table 4, the sensitivity and specificity were 0.914 and 0.898, respectively. The accuracy was 0.898 (95% CI: 0.881, 0.913), and the corresponding value of YI was 0.036.

### 3.4. Performance of C&RT

The ROC curve obtained by the C&RT model is shown in Figure 2. The AUROC was 0.682, and the accuracy was 0.978 (95% CI: 0.968, 0.985). The sensitivity and specificity were 0.371 and 0.992, respectively (Table 4). The corresponding value of YI was 0.360.

### 3.5. Performance of SVMs

The ROC curve obtained by the SVM model is shown in Figure 2. The AUROC was 0.765, and the accuracy was 0.919 (95% CI: 0.966, 0.983). The sensitivity and specificity were 0.571 and 0.928, respectively (Table 4). The corresponding value of YI was 0.039.

### 3.6. Performance of RF

The ROC curve obtained by RF is shown in Figure 2. The AUROC was 0.949, and the accuracy was 0.843 (95% CI: 0.823, 0.862). The sensitivity and specificity were 0.971 and 0.840, respectively (Table 4). The corresponding value of YI was 0.035.

### 3.7. Performance of GBM

The ROC curve obtained by GBM model is shown in Figure 2. The AUROC was 0.952, and the accuracy was 0.883 (95% CI: 0.866, 0.900). The sensitivity and specificity were 0.943 and 0.881, respectively (Table 4). The corresponding value of YI was 0.034.

### 3.8. Comparison of the AUROC of Prediction Models

As shown in Figure 2, although the AUROC of the GBM was the largest, there was no statistically significant difference between the GBM, RF, and MLR models. Moreover, no statistically significant difference was found between CART and SVM models. Therefore, the AUROC of these models was as follows: GBM = RF = MLR > CART = SVM. The details of pairwise comparisons of the *P* values are shown in Table 5.

## 4. Discussion

**Figure 3:**
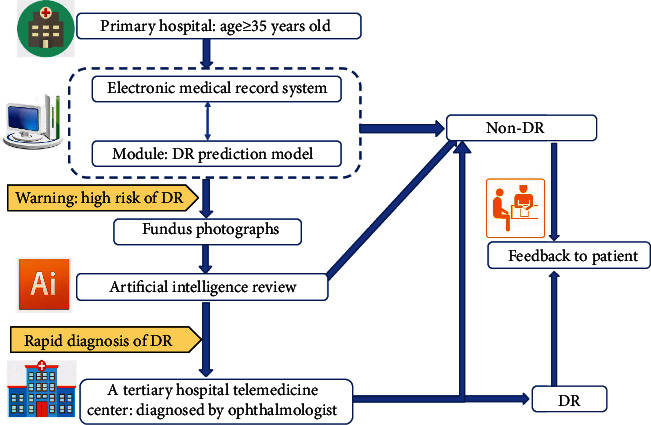
The procedure of DR diagnosis in primary hospital.

In order to find the optimal DR prediction model, the current study established and compared several prediction models including the traditional statistic algorithms, ensemble learning algorithms, and basic machine learning algorithms. Five DR prediction models in the Chinese rural population were established in the current study, and the performance of these models was compared. In the early stage of DR, there may be no visual symptoms. If DR patients are identified and diagnosed early, irreversible visual impairment could be prevented. Conversely, if not, the damage will be irreversible as the DR continues to advance [38, 39]. In China, the rural residents accounting for more than half (64%) of the total population, and there were significant regional differences in health resources allocation between the city and the rural area, with the underdeveloped areas having access to fewer health resources [40, 41]. Early detection, diagnosis, and treatment are vital in preventing such irreversible damage, especially in less-developed rural areas. With the development of technology in DR diagnosis through artificial intelligence (AI) on eye fundus photographs, DR patients can be initial diagnosis easily. Moreover, more and more level A tertiary hospitals have set up telemedicine centers to collaborate with the primary hospitals on the diagnosis of DR, which means that rural DR patients can get a final diagnosis in the primary hospitals. Developing the DR prediction model based on the data collected by the primary hospitals in the rural areas allows the identification of the patients with high risk for DR, then makes a rapid diagnosis for those patients through AI fundus photograph review. Subsequently, the final diagnosis could be made via telemedicine center services (Figure 3). This procedure can be used to screen the majority of rural DR patients in the primary hospitals, which can not only save the cost of economic expenses and human resources but also improve the detection rate of DR in China. Therefore, it is of certain public health significance and practical value to develop the prediction model.

In the current study, the MLR model and machine learning algorithms were used to establish the prediction model based on the data of HES. The GBM, RF, and MLR models showed excellent prediction performance on DR, with an AUROC of 0.952, 0.949, and 0.936, respectively, which were significantly larger than that of SVM and CART. However, the accuracy of CART and SVM was higher than that of GBM, RF, and MLR. Theoretically, the AUROC is more robust than accuracy when evaluating and comparing classifiers, especially based on the skew distribution samples [42]. In this study, the proportion of DR patients was 2.5%. Therefore, the prediction performance of these models would depend on the value of AUROC.

The good prediction performance of the GBM and RF might be due to the fact that both models used “ensemble learning” algorithms. In nature, ensemble learning is to train multiple “weak learners” as members of it and combine their predictions into a single output, thereby making accurate predictions. The CART and SVM models are “weak learners” also referred to as “base learners”. The study showed that the AUROC of both CART and SVM models was below 0.8, which was consistent with previous studies that the prediction performance of ensemble learning models is always better than weak learning models [43, 44].

Moreover, the prediction performance of the MLR model was similar to that of the GBM and RF and better than SVM and CART algorithms. Stylianou et al. have indicated in their study that the logistic regression model has better prediction performance on the mortality risk of burn injury patients than machine learning algorithms [45], and a similar conclusion also can be found in other studies [46, 47]. Actually, there was no substantial association between the algorithm complexity and prediction performance of DR found in this study. Furthermore, the MLR allows the visualization of the modeling process of prediction and provides the critical predicted factors as well as the value of predict factors. To sum up, the MLR model was the optimal model on the DR early diagnosis owing to its excellent prediction performance and visible equation.

According to the fitting result of the MLR model, blood glucose was independently associated with DR in this study. The correlation coefficient between blood glucose and the probability of DR in this study was over 0.9. It has been verified in many studies that poor glycemic control has a high positive correlation with DR occurrence among diabetes patients. Therefore, it is critical for diabetic patients to continuously control their blood glucose concentrations so as to avoid the occurrence of DR. Additionally, the SBP was correlated with the development of DR. Many studies have confirmed the association between blood pressure (BP) and DR [48, 49]. One of the evidence-supported pathogenesis is that the higher BP would induce increased expression of vascular endothelial growth factor (VEGF), thereby leading to the development of DR [50, 51]. The other theory is that the higher BP leads to hemodynamic alternations, including microvascular damage, abnormal lipid metabolism, and hemorheology changes in retinal microvasculature, thereby aggravating the microangiopathy of retinal [52]. Therefore, rational control of hypertension is important to slow down the microangiopathy in diabetic patients.

In addition, the BMI exhibited a positive correlation with DR occurrence. The relationship between BMI and DR has been similarly confirmed by many epidemiologic studies [53–55]. The pathogeneses proposed for this association include metabolic syndrome [56] and increased oxidative stress [57]. Nonetheless, there are also contradictory findings. Klein et al. proposed in their study that a higher BMI confers a protective effect on DR in Asian type 2 diabetic patients, while a higher WHR is associated with the DR in women. The Wisconsin Epidemiologic Study of Diabetic Retinopathy found that the association between obesity and DR is limited only to individuals with older-onset insulin-independent diabetes. Other studies found that decreased BMI is associated with a higher prevalence of DR in white populations [58, 59]. The differences in the findings of the above studies may be attributable not only to genetic differences like racial and ethnic differences but also to the demographic difference.

This study also has some limitations. Although the MLR model showed good prediction performance, there were only three significant prediction factors. Moreover, there might be an inevitable bias caused by the patients' loss ofof follow-up. In addition, since the correlation coefficient between blood glucose and DR in this study was over 0.9, this would affect the other potential factors like total triglycerides, high-sensitive C-reactive protein, and urea albumin included in the MLR model. Furthermore, this study lacks a test set, which should be a similar, independent population, for the evaluation of the generalization capability of the final model, and this would limit the extrapolation of the DR prediction model.

In conclusion, this study used the data of HES, which focuses on the rural adult population in China. Given the limited ophthalmic resources in rural areas, the prediction model has the potential to serve as a complementary practical screening tool for the early detection of DR, especially for diabetic patients in remote areas. Moreover, the MLR model was the optimal model on the DR early diagnosis owing to its excellent prediction performance and visible equation.

## Figures and Tables

**Table 1:** Demographic characteristics and univariate analysis (*n* = 4572).

Variable	DR^※^	Non-DR^※^	Statistic value	*P* value
Age	54.00 (50.00-59.00)	52.00 (42.00-58.00)	3.778^∗^	<0.001
Gender			1.434^#^	0.231
(i) Male	47 (39.50%)	2086 (45.02%)		
(ii) Female	72 (60.50%)	2547 (54.98%)		
Education degree			4.866^#^	0.088
(i) Illiterate or semi-illiterate	14 (11.76%)	619 (13.36%)		
(ii) Primary grade	73 (61.34%)	2376 (51.28%)		
(iii) Secondary school and above	32 (26.89%)	1638 (35.36%)		
Marriage status			6.645^#^	0.010
(i) Married	19 (15.97%)	419 (9.04%)		
(ii) Divorced and widowed	100 (84.03%)	4214 (90.96%)		
Smoke status			0.849^#^	0.357
(i) No	86 (72.27%)	3164 (68.29%)		
(ii) Yes	33 (27.73%)	1469 (31.71%)		
Drink status			0.025^#^	0.876
(i) No	94 (78.99%)	3632 (78.39%)		
(ii) Yes	25 (21.01%)	1001 (21.61%)		
Systolic blood pressure	143.00 (130.00-164.50)	134.50 (122.00-150.50)	4.232^∗^	<0.001
BMI	25.63 (22.68-28.44)	24.12 (22.07-26.37)	3.978^∗^	<0.001
Spheric equivalence	0.13 (-0.50-0.63)	0.00 (-0.50-0.50)	0.917^∗^	0.359
IOP	16.00 (14.25-17.75)	15.00 (13.00-17.00)	3.853^∗^	<0.001
Axis length	22.68 (22.16-23.22)	22.81 (22.30-23.30)	-1.822^∗^	0.068
Blood glucose	6.88 (6.13-9.11)	5.49 (5.14-5.89)	12.503^∗^	<0.001
Urea nitrogen	4.51 (3.93-5.53)	4.65 (3.93-5.49)	-0.281^∗^	0.779
Creatine	69.80 (63.60-78.90)	69.70 (63.60-77.20)	0.415^∗^	0.679
Total triglycerides	1.55 (1.06-2.28)	1.27 (0.88-1.85)	3.729^∗^	<0.001
High-sensitive C-reactive protein	2.13 (0.79-4.38)	1.19 (0.43-3.33)	3.181^∗^	0.002
Albumin	45.50 (43.00-48.00)	45.00 (42.93-47.00)	1.674^∗^	0.094
Urea creatinine	75.20 (41.90-118.90)	75.20 (46.40-114.55)	-0.474^∗^	0.636
Urea albumin	1.28 (0.41-5.64)	0.71 (0.31-2.12)	3.102^∗^	0.002
Uric acid	1211.00 (833.00-1895.00)	1222.00 (827.00-1709.30)	0.578^∗^	0.563

Note: ※ = continuous variables were showed as average and range; categorical variables were showed as percentage. ^∗^ = the Wilcoxon rank sum test. # = chi-squared test.

**Table 2:** The kappa coefficient between two ophthalmologists.

Doctor Hu	Doctor Zhang		Kappa
	DR	Non-DR	
DR	108	13	0.857
Non-DR	22	4609	

Note: Doctor Hu is referring to Ailian Hu; Doctor Zhang is referring to Xu Zhang.

**Table 3:** Model performance using multivariable logistic regression model (*n* = 4572).

Variable	Standardized regression coefficient	*P* value
Intercept	-12.646	<0.001
Age	0.004	0.756
Marriage status	-0.385	0.286
Systolic blood pressure	0.011	<0.05
BMI	0.078	<0.05
IOP	-0.009	0.836
Blood glucose	0.966	<0.001
Total triglycerides	-0.157	0.231
High-sensitive C-reactive protein	0.009	0.691

Note: BMI: body mass index; IOP: intraocular pressure.

**Table 4:** Performance of validation of the DR prediction models.

Model	Accuracy (95% CI)	Sensitivity	Specificity
GBM	0.883 (0.866, 0.900)	0.943	0.881
RF	0.843 (0.823, 0.862)	0.971	0.840
MLR	0.898 (0.881, 0.913)	0.914	0.898
SVMs	0.919 (0.966, 0.983)	0.571	0.928
C&RT	0.978 (0.968, 0.985)	0.371	0.992

Note: Gradient boosting machine (GBM); random forests (RF); multivariable logistic regression (MLR); support vector machine (SVM); classification and regression trees (C&RT).

**Table 5:** The details of pairwise comparisons of the *P* values.

Model	GBM	RF	MLR	SVM	C&RT	AUROC
GBM	—	0.624	0.408	<0.001	<0.001	0.952
RF	0.624	—	0.538	<0.001	<0.001	0.949
MLR	0.408	0.538	—	<0.001	<0.001	0.936
SVM	<0.001	<0.001	<0.001	—	0.052	0.765
C&RT	<0.001	<0.001	<0.001	0.052	—	0.682

Note: Gradient boosting machine (GBM); random forests (RF); multivariable logistic regression (MLR); support vector machine (SVM); classification and regression trees (C&RT).

## Data Availability

The data set analyzed in the current study is not publicly available as it contains private patient data from HES. The information excluding patient identification and demography is available upon request for research purpose.

## Additional Points

*Highlights*. (i) The current study is the first one that identifies the optimal model for the prediction of diabetic retinopathy in the Chinese rural population by comparing the prediction performance of traditional statistic algorithms and machine learning algorithms. (ii) Our findings are based on the Handan Eye Study which is a longitudinal study design with a large sample size. Therefore, the study has the potential to serve as a practical screening tool for the early detection of DR, especially in the rural areas of China. (iii) This study updates the evidence in the field, which provides a scientific basis for future research directed toward DR prediction, especially through machine learning algorithms.
